# Endometrial cancer molecular classification: research advances and clinical application

**DOI:** 10.3389/fonc.2026.1947939

**Published:** 2026-10-05

**Authors:** Ya Liu, Yueying Li, Liping Zhang

**Affiliations:** 1 Department of Obstetrics and Gynecology, Wuhan Children’s Hospital Graduate Joint Training Base, School of Medicine, Wuhan University of Science and Technology, Wuhan, China; 2 Department of Obstetrics and Gynecology, Tianjin Medical University General Hospital, Tianjin, China; 3 Department of Gynecology, Wuhan Children’s Hospital (Wuhan Maternal and Child Healthcare Hospital), Tongji Medical College, Huazhong University of Science & Technology, Wuhan, China

**Keywords:** clinical translation, endometrial cancer, molecular classification, precision medicine, prognosis, treatment prediction

## Abstract

As research in molecular biology continues to advance, the classification of endometrial cancer has gradually shifted from traditional histology toward risk stratification based on the integration of molecular subtyping with clinicopathologic factors. Molecular subtyping has not only transformed our understanding of the biological heterogeneity of endometrial cancer but also provided new evidence for prognostic assessment and treatment selection. However, the maturity of clinical evidence and the translational therapeutic value vary across molecular subtypes. This review systematically summarizes the molecular characteristics and clinical significance of the POLE ultramutated(POLE-mut)subtype, mismatch repair deficient (dMMR) subtype, p53 abnormal (p53-abn) subtype, and no specific molecular profile (NSMP) subtype, with a focus on evaluating whether different molecular features can be further translated from risk stratification into specific treatment decisions. Centering on key issues such as de-escalated therapy for the POLE-mut subtype, the predictive value of immunotherapy for the dMMR subtype, intensified therapy for the p53-abn subtype, and further risk stratification of the NSMP subtype, this review analyzes the clinical basis and limitations of existing evidence and further discusses the challenges that tumors with multiple molecular features pose for classification and treatment decision making. Building on this, the paper compares how major international guidelines integrate molecular information with clinical and pathologic factors and analyzes real world implementation barriers such as testing standardization, result interpretation, and healthcare accessibility. Finally, it outlines the potential value of emerging technologies, including liquid biopsy, artificial intelligence, and multiomics, in dynamic risk assessment and precision therapy. This review aims to trace the continuum of evidence from molecular discovery to clinical action, clarifying which clinical decisions can be supported by existing molecular markers and identifying questions that current evidence cannot yet answer, thereby providing guidance for the precise application and clinical translation of molecular subtyping in endometrial cancer.

## 1 Introduction

Endometrial cancer (EC) is a gynecologic malignancy characterized by substantial biological heterogeneity, and its incidence is increasing worldwide. According to the Global Cancer Statistics Report, in 2024, there were 434,620 new cases of cancer of the uterine corpus, primarily endometrial cancer and including a small number of uterine sarcomas, among women worldwide, ranking 16th among 34 cancer types. There were 100,680 deaths, ranking 18th (1). It is estimated that in 2026, there will be 68,270 new cases of corpus uteri cancer in the United States, with 14,450 deaths. From 2013 to 2022, the incidence of endometrial cancer in the United States increased from 26.4 per 100,000 to 28.8 per 100,000, while the mortality rate increased from 4.8 per 100,000 to 5.6 per 100,000 from 2014 to 2023 (2).

The development of endometrial cancer (EC) results from the interplay of multiple factors. Obesity is a major risk factor that affects hormonal metabolism and the biological characteristics of EC (3). Insulin resistance and hyperglycemia are also significant risk factors, as they can directly promote cell proliferation and survival through the PI3K/Akt and Ras/MAPK signaling pathways (4). Both environmental and genetic factors play important roles. Exposure to endogenous and exogenous estrogens is a major environmental risk factor (5). In addition, genetic factors such as Lynch syndrome are associated with a significantly increased lifetime risk of EC and an earlier age at onset (6).

Various factors influence the clinical outcomes of endometrial cancer (EC). In addition to tumor type and grade, clinical and pathologic factors such as age, tumor stage, and lymphovascular space invasion are also used for risk assessment (7) and are closely associated with molecular subtyping (8). In recent years, as research in this field has advanced, risk stratification based on molecular characteristics has gradually transformed our understanding of the biological behavior of endometrial cancer and driven a shift in disease management from an empirical, morphology based diagnostic and therapeutic model toward precision risk stratification that integrates molecular information (9). This shift has extended beyond the research setting: the FIGO 2023 staging system introduced molecular subtype adjusted staging (10), and the 2025 ESGO-ESTRO-ESP guidelines further emphasized the integration of molecular subtyping with traditional clinical and pathologic factors (11).

However, the application of molecular subtyping in clinical practice does not imply that all molecular subtypes have the same value in guiding treatment (11). Current evidence indicates that the prognostic characteristics of various molecular subtypes are relatively well defined. However, prognostic relevance does not directly equate to the ability to predict benefit from specific treatments. Specifically, the POLE-mut subtype is associated with a relatively favorable prognosis, but the applicability and long term safety of deescalated therapy still require further validation in prospective studies (12–14). The dMMR subtype has become an important predictor of response to immune checkpoint inhibitors and has substantially influenced treatment selection for some patients with advanced or recurrent disease (15, 16). The p53-abn subtype typically exhibits aggressive biological behavior and poor prognosis. However, evidence on how to optimize intensified treatment strategies based on this molecular characteristic remains insufficient (12). The NSMP subtype, due to its high degree of internal heterogeneity (17, 18), currently lacks a unified and clearly defined molecularly guided treatment pathway. In summary, molecular subtyping has largely addressed the identification of distinct biological subgroups. However, how to further translate molecular information into clear and differentiated treatment strategies remains a key challenge in current clinical translation.

The current molecular classification system and its clinical translation process still face constraints at multiple levels. At the evidence level, there is no linear relationship between prognostic relevance, predictive value for treatment, and changes in clinical practice. Furthermore, different sources of evidence, including prospective and retrospective cohorts, clinical trials, and case control studies, vary in terms of evidence level and generalizability (12, 15, 16). At the biological level, existing classification systems struggle to fully capture the complexity of tumor biology, including intramolecular subtype heterogeneity within the NSMP subtype (11), the classification hierarchy of tumors with multiple molecular features (19), and the dynamic evolution of intratumoral heterogeneity and molecular subtyping as the disease progresses and undergoes treatment. Furthermore, practical issues such as testing standardization, cost, and access to healthcare resources (20) may also pose barriers to the clinical translation of molecular subtyping. Against this backdrop, it is crucial to determine whether emerging technologies (21) can address the limitations of existing classification systems in areas such as dynamic monitoring, analysis of intramolecular subtype heterogeneity, and testing accessibility and ultimately be translated into practical tools to improve clinical decision making.

Therefore, this review adopts the perspective of translating EC molecular subtyping into clinical practice. Building on an overview of the evolution of molecular subtyping systems, it focuses on the clinical significance of different molecular subtypes and the evidence supporting subtype specific treatment, while further analyzing the limitations of existing classification systems in terms of biological heterogeneity, multiple molecular features, and clinical implementation. We evaluate the practical value of different molecular subtypes in clinical decision making and explore the potential contributions of emerging technologies to molecular subtyping systems. Through this lens, we aim to provide guidance for the further translation of EC molecular subtyping from risk stratification to precision clinical decision making.

This review is a narrative review; therefore, no independent two researcher literature screening or risk of bias assessment was conducted. To enhance the transparency and reproducibility of the literature search and screening process, we established a search protocol. A comprehensive literature search was conducted using databases such as PubMed, Web of Science, Embase, and Scopus, with the search limited to articles published through August 2026. Search keywords included terms such as endometrial cancer, molecular subtyping, TCGA, ProMisE, precision medicine, and prognostic outcomes. We included original research articles published in English, including prospective and retrospective cohort studies, case control studies, randomized controlled trials, and animal studies, as well as systematic reviews and meta analyses. Conference abstracts, isolated case reports, and preprints that had not yet been formally published were excluded. Literature screening was conducted independently by one researcher, who first screened titles and abstracts, followed by full text review for eligibility. Priority was given to landmark original studies, such as those from TCGA and ProMisE, large sample clinical cohort studies, highly cited authoritative reviews, and the latest studies on clinical trials and molecular testing technologies. When conflicting evidence was identified, conclusions from large sample, high quality studies were given priority.

## 2 From traditional pathology to molecular subtyping: the evolution of classification systems

### 2.1 Traditional clinical pathology classification systems and their limitations

For many years, clinical risk stratification for EC has primarily relied on the Bokhman dual model and the WHO histopathological classification system. Based on the Bokhman dual model, EC has historically been classified into Type I, estrogen related and endometrioid, and Type II, nonestrogen related and nonendometrioid (22). Type I EC typically presents with an endometrioid histological pattern, well differentiated tumors, early diagnosis, and a relatively favorable prognosis, whereas Type II EC is primarily characterized by high grade nonendometrioid histology, aggressive invasion, and poorer long term survival (22). This model provides preliminary insights into the distinct biological behaviors of the two types. The WHO classification system further subdivides EC into subtypes such as endometrioid carcinoma, serous carcinoma, clear cell carcinoma, and carcinosarcoma based on histomorphology. Combined with histological grading, depth of myometrial invasion, lymphovascular space invasion, and FIGO staging, this system forms a postoperative risk stratification framework that has long served as the core basis for clinical treatment decision making (23).

However, as clinical data have accumulated and molecular biology research has advanced, the inherent limitations of traditional pathologic classification systems have gradually become apparent. First, the Bokhman dual type model is a conceptual hierarchical model that encompasses a substantial number of borderline and overlapping cases. Some high grade endometrioid cancer exhibit both the histological features of Type I and the aggressive biological behavior of Type II, making them difficult to clearly classify within a binary framework (24). Rare histological types, such as clear cell carcinoma and carcinosarcoma, exhibit substantial molecular and phenotypic heterogeneity and cannot be fully categorized as either Type I or Type II (25). Second, interobserver agreement in morphologic diagnosis is limited. In particular, substantial differences in pathologic interpretation have been reported when distinguishing high grade endometrioid cancer from serous cancer, which directly affects prognostic assessment and treatment selection. Multiple studies have demonstrated that even among experienced gynecologic pathologists, the consistency of histological grading for endometrioid cancer is only moderate, with κ values ranging from 0.41 to 0.65 (26). Furthermore, tumors with identical histological morphology may have markedly different prognostic outcomes. Some low grade endometrioid cancers present with early distant metastasis (27), whereas a small proportion of high grade cases demonstrate an indolent course (28). A risk stratification system that relies solely on morphologic classification is prone to bias in prognostic prediction and has limited ability to achieve precise, individualized risk assessment. A more fundamental limitation is that traditional classification systems lack predictive biomarkers to guide the selection of targeted therapies. As current research has entered the era of immunotherapy and various new drugs have entered clinical practice, this shortcoming has become increasingly apparent. A treatment decision making model without molecular biomarker guidance can no longer fully meet the demands of precision medicine (29). These shortcomings have driven the transition of the EC classification system from one based solely on morphology toward a comprehensive, precision based classification approach that integrates clinicopathologic features with molecular subtyping.

### 2.2 History of molecular subtyping in endometrial cancer

In 2013, based on the findings of The Cancer Genome Atlas (TCGA) study, EC was classified into four molecular subtypes: the POLE ultramutated subtype(POLE-mut subtype), the microsatellite instability hypermutated subtype(MSI-H subtype), the copy number low subtype(CNL subtype), and the copy number high subtype (CNH subtype) (8). These subtypes not only differ in their genetic characteristics but also exhibit substantial differences in clinical prognosis and treatment response (8, 30). This study drove the transition of the EC classification system from a traditional morphologic model to a molecular classification model and laid the theoretical foundation for more precise tumor risk stratification based on molecular characteristics (30). Although the TCGA classification system is widely recognized for its strong prognostic value, its clinical application faces numerous challenges because of its technical complexity (8, 9). This has spurred the development of clinically practical classification methods based on immunohistochemistry (IHC) and next generation sequencing (NGS), such as the Proactive Molecular Risk Classifier for Endometrial Cancer (ProMisE). The ProMisE protocol classifies cases into three molecular subtypes: the POLE ultramutated subtype(POLE-mut), the mismatch repair deficient subtype (dMMR, corresponding to the MSI-H subtype), and the p53 abnormal subtype(p53-abn, corresponding to the CNH subtype). Cases without any of these three molecular alterations are classified as having a no specific molecular profile (NSMP, corresponding to the CNL subtype) (9). This method has been further validated in a large, population based independent cohort and has demonstrated consistent classification and prognostic stratification capabilities, supporting the broader adoption of personalized prognostic assessment and treatment selection (31). This evolution from the TCGA research findings to the clinically implementable ProMisE system marks the successful translation of EC molecular subtyping from basic research to clinical application, establishing its pivotal role in linking tumor biology, risk stratification, and personalized therapy.

## 3 Biological characteristics and clinical significance of the four molecular subtypes

### 3.1 POLE-mut subtype

The POLE-mut subtype driven EC is primarily driven by pathogenic somatic mutations in the exonuclease domain of the *POLE* gene, resulting in impaired DNA proofreading, a markedly elevated mutation burden, and a strong antitumor immune response (32). This subtype accounts for approximately 5% to 10% of all endometrial cancers, with an even higher proportion observed among high grade endometrioid cancers (32, 33).

Current evidence generally indicates that the POLE-mut subtype has distinctive molecular characteristics and a favorable prognosis. This prognostic advantage has been reported across different histological types and staging contexts, although long term follow up data remain limited. Meng et al. found that, among patients with grade 3 EC, mutations in the *POLE* exonuclease domain were associated with an extremely low risk of disease progression. None of the 8 patients with the POLE-mut subtype in the cohort experienced disease progression during follow up, and multivariate analysis also indicated that POLE-mut subtype were an independent protective factor (p = 0.010) (34). Another cohort study involving 381 patients with stage 3 EC showed that the 5 year recurrence free survival rate among patients with the POLE-mut subtype reached 96%, which was significantly higher than that of other molecular subtypes (35). However, the POLE-mut subtype is not entirely homogeneous, and its clinical outcomes may still be influenced by the mutation site, coexisting molecular alterations, histological type, and other pathologic factors. Importantly, not all POLE variants are pathogenic. Only pathogenic mutations that clearly affect the function of the exonuclease domain have value for molecular subtyping (32, 33). Therefore, although the POLE-mut subtype generally exhibits a high mutation burden and a favorable prognosis, a certain degree of internal heterogeneity remains. Prognostic assessment in individual cases should therefore be based on a comprehensive evaluation of molecular and clinicopathologic characteristics (14, 36).

### 3.2 dMMR subtype

The molecular basis of the dMMR subtype and MSI-H subtype endometrial cancer lies in germline mutations or epigenetic silencing of mismatch repair genes, such as *MLH1, MSH2, MSH6*, and *PMS2*, leading to their inactivation. This in turn disrupts the DNA mismatch repair system, allowing replication errors in microsatellite sequences to accumulate and ultimately resulting in microsatellite instability and a high mutation burden (37). This subtype accounts for approximately 25% to 30% of all endometrial cancers (38). Notably, the underlying mechanisms of the dMMR subtype follow a dichotomous pattern. The majority of sporadic cases are driven by methylation of the *MLH1* promoter region, whereas a small proportion is caused by germline mutations in mismatch repair genes, as seen in Lynch syndrome. This naturally links MMR testing to the identification of hereditary tumors (39).

Multiple studies suggest that the dMMR/MSI-H subtype EC has relatively favorable disease-specific survival and a moderate prognosis (40, 41). More importantly, there is significant prognostic heterogeneity within the dMMR subtype. Different mechanisms of MMR deficiency result in markedly different clinical outcomes for the dMMR subtype EC: sporadic cases driven by *MLH1* promoter methylation exhibit a higher tendency toward recurrence, whereas Lynch-associated dMMR tumors arising from germline mutations have a relatively better prognosis. This suggests that prognostic heterogeneity within the dMMR subtype is driven by different molecular mechanisms and that this subtype should not be regarded as a homogeneous group (12, 42). A study involving 899 patients with the dMMR subtype EC showed that, after stratification by MMR protein loss pattern, the MLH1-deficient group (80%) had significantly higher risks of recurrence and death than the MSH2/MSH6/isolated PMS2-deficient group (20%), suggesting that tumors driven by different inactivated MMR genes exhibit heterogeneous clinical outcomes (43). This heterogeneity suggests that classifying dMMR/MSI-H subtype as a single prognostic category is insufficient to adequately reflect the differences in outcomes within this subtype; on the contrary, it may compromise the accuracy of clinical risk stratification.

### 3.3 p53-abn subtype

The core molecular event in the p53-abn subtype is a *TP53* gene mutation, accompanied by widespread somatic copy number alterations and chromosomal instability. Histologically, this subtype typically corresponds to serous cancer or high grade endometrioid cancer (8). The TCGA study found that serous endometrial cancer and approximately 25% of high grade endometrioid cancers exhibit extensive copy number abnormalities, limited DNA methylation changes, a high frequency of *TP53* mutations, and low estrogen and progesterone receptor expression, suggesting that this subtype is characterized by chromosomal instability and aggressive biological behavior distinct from that of typical endometrioid cancer (8). In clinical practice, abnormal p53 immunohistochemical expression patterns, including overexpression or complete loss, are widely used as surrogate markers for the p53-abn molecular subtype. However, it is important to note that p53-abn is not synonymous with *TP53* mutations. Rather, it is essentially a functional definition of this subtype based on protein expression patterns rather than direct genotyping (9, 31).

The most definitive clinical value of this subtype lies in its consistently poor prognostic significance. In the PORTEC-3 trial, an analysis of high risk endometrial cancer molecular subgroups revealed that the 5 year recurrence free survival (RFS) rate among patients with p53-abn was only 48%, significantly lower than that of other subtypes (12). The poor prognosis associated with the p53-abn subtype is consistent across different FIGO stages and is independent of traditional histological grading (12, 35).

### 3.4 NSMP subtype

The NSMP subtype refers to a molecular category defined after excluding pathogenic POLE-mut subtype, mismatch repair defects, and p53 abnormalities, corresponding to the copy number low subtype in the TCGA classification (9). The NSMP subtype accounts for approximately half of all endometrial cancers and has an overall prognosis intermediate between those of the POLE-mut and p53-abn subtypes (9). However, this overall classification as an intermediate risk group masks substantial internal clinical, histological, and genomic heterogeneity (44). A large scale genomic study of 472 patients with NSMP subtype EC found that these tumors could be further divided into multiple genomic clusters with distinct alterations involving *PTEN*, *PIK3CA/PIK3R1*, and chromosome *1q*. Among these, the subgroup characterized by high level 1q gain and absence of *PTEN* mutations more frequently presented with high grade histology, low ER expression, and advanced disease and had a poorer prognosis, suggesting that NSMP is not a single biological entity in the strict sense (44). These findings have driven a shift in NSMP subtype research from viewing it as a remaining category within the four tier classification toward further internal risk stratification.

The heterogeneity of the NSMP subtype is primarily reflected in the lack of uniformity in its underlying molecular pathways. Unlike the p53-abn subtype, which is characterized by chromosomal instability, the NSMP subtype generally exhibits a relatively stable copy number profile and frequently harbors mutations in the PI3K/AKT pathway, including alterations in *PTEN*, *PIK3CA*, and *PIK3R1*. However, substantial differences still exist among different genomic clusters (44). Among these alterations, *CTNNB1* mutations can lead to abnormal stabilization and nuclear accumulation of β-catenin, thereby resulting in persistent activation of the Wnt/β-catenin signaling pathway. This represents a potential molecular driver that has attracted considerable attention in NSMP subtype. Previous studies and systematic reviews suggest that, particularly in early stage endometrioid cancer, *CTNNB1* exon 3 mutations may be associated with an increased risk of recurrence (45). However, findings from recent studies have not been entirely consistent. In 2025, a large scale meta analysis by Ferrari et al. involving 4,654 patients with the NSMP subtype did not demonstrate an independent prognostic significance of *CTNNB1* mutations (46). Therefore, *CTNNB1* mutations are currently better regarded as a biologically relevant marker for further stratification of NSMP, and their prognostic value remains an evidence gap requiring prospective validation.

Compared with *CTNNB1*, hormone receptor status, particularly ER status, is one of the biomarkers with the most well established evidence for internal subclassification of the NSMP subtype. In the TransPORTEC analysis of 648 cases of high risk endometrial cancer, ER positivity was associated with a lower risk of recurrence among patients with NSMP, and within the high risk NSMP subgroup, ER positivity was an independent predictor of a lower risk of recurrence (HR 0.33, 95% CI 0.15 to 0.75) (47). Subsequently, Jamieson et al. analyzed 1,110 cases of NSMP endometrial cancer and found that histological grade and ER status could identify a subgroup with an extremely low risk of disease specific mortality. The 5 year disease specific mortality rate among patients with G1 to 2 and ER positive NSMP was only 1.6%, whereas it increased to 22.9% among patients with G3 and/or ER negative tumors (48). Large scale meta analyses by Lam et al. and Ferrari et al. further validated the prognostic predictive value of ER (46, 49). Therefore, ER status may be one of the most promising simple, reproducible, and clinically implementable indicators currently available for further stratifying the NSMP subtype into low risk and high risk groups. The absence of PR also shows a trend toward an association with poorer outcomes, but the current evidence is less consistent, and its independent predictive ability is weaker than that of ER (46, 47, 49).

As a cell adhesion molecule, L1CAM represents another important aspect of internal stratification within NSMP. Its abnormal expression is closely associated with disease progression. Within the ProMisE classification framework, L1CAM positivity is particularly useful for further identifying high risk subgroups among p53 wild type and NSMP subtype patients (50, 51). A recent large scale meta analysis involving 4,654 patients with NSMP showed that L1CAM overexpression was significantly associated with a higher risk of recurrence (HR 3.42) and poorer survival (HR 4.31) (46). However, L1CAM is currently used primarily for prognostic prediction, and its ability to independently guide the selection of adjuvant therapy has yet to be prospectively validated. Therefore, it should not be considered equivalent to established predictive biomarkers for treatment response.

*ARID1A* and *KRAS* also reflect the internal heterogeneity of NSMP. *ARID1A* is a core component of the SWI/SNF chromatin remodeling complex and is relatively common in NSMP. A retrospective study showed that *ARID1A* mutations in NSMP endometrial cancer are associated with a higher risk of recurrence and poorer PFS (52). A Chinese NSMP subtype cohort study also found that *ARID1A* mutations were associated with poorer PFS and frequently co occurred with *PIK3CA* mutations (53). *KRAS* is a potential driver alteration of interest in NSMP genomics. A study involving 7,870 cases of endometrial cancer showed that *KRAS* mutated tumors exhibit distinct genomic characteristics and may overlap with biomarkers associated with immunotherapy. However, current evidence is insufficient to demonstrate that *KRAS* can independently stratify the prognosis of NSMP subtype or guide specific treatment selection (54). Therefore, *ARID1A* and *KRAS* are currently better regarded as research directions for investigating the biological heterogeneity of NSMP subtype and potential therapeutic targets rather than as established criteria for clinical subtyping.

In recent years, markers associated with the immune microenvironment have also been incorporated into studies on the subclassification of the NSMP subtype. A study of 208 cases of endometrial cancer found that HLA-I deficiency was associated with high grade tumors, deep myometrial invasion, marked lymphovascular space invasion, and an immune rejection phenotype. Moreover, its adverse prognostic impact was primarily observed in the NSMP subtype. In patients with early stage NSMP subtype, HLA-I deficiency independently predicted poorer disease free survival (55). These findings suggest that heterogeneity within the NSMP subtype stems not only from genetic alterations in tumor cells themselves but may also arise from interactions between antigen presentation and the tumor immune microenvironment. However, as this field remains in the early stages of validation, HLA-I deficiency is currently better regarded as a potential marker of immune evasion and risk stratification rather than as a direct clinical predictor of resistance to immunotherapy.

Overall, the stratification of the NSMP subtypes is gradually shifting from the evaluation of individual markers toward integrated multivariable models. Existing studies have demonstrated that characterizing patients solely by their NSMP subtype may obscure a continuous spectrum of risk ranging from extremely low to markedly high. However, to date, these markers within NSMP subtype lack sufficient prospective validation and cannot yet directly guide treatment decisions as independent molecular subtypes comparable to the POLE-mut subtype, dMMR subtype, or p53-abn subtype. Therefore, the main challenge in NSMP subtyping is not a lack of candidate markers, but rather whether prospective studies can confirm that these markers can further improve the accuracy of risk stratification and ultimately inform treatment decisions that improve clinical outcomes.

### 3.5 Multiple-classifier endometrial cancer

The four molecular subtype classification system for endometrial cancer established by the TCGA and implemented through the ProMisE framework was initially designed such that each tumor could be assigned to one mutually exclusive molecular category. This classification approach has been widely incorporated into risk stratification models, pathology reports, and clinical practice guidelines (56). However, multiple studies have shown that a small proportion of cases may simultaneously exhibit two or more molecular features, constituting multiple-classifier EC (19, 57). The most commonly observed combinations in clinical practice are POLE-mut plus p53-abn subtypes, dMMR plus p53-abn subtypes, and POLE-mut plus dMMR subtypes. In addition, a very small number of cases exhibit triple molecular alterations, such as POLE-mut plus dMMR plus p53-abn subtypes (57).

Although endometrial cancers with multiple molecular features account for a relatively small proportion of the overall population, the differences they exhibit in treatment decision making and prognostic outcomes are clinically significant. Current evidence suggests that different combination patterns may reflect distinct biological mechanisms. The clinical behavior of the POLE-mut subtype and p53-abn subtype tumors tends to align with that of POLE-mut subtype tumors rather than p53-abn subtype tumors. A study involving 14 patients showed that even among advanced POLE-mut subtype cases with abnormal p53 expression, half of the patients maintained favorable clinical outcomes, consistent with the dominant prognostic role of the POLE-mut subtype (58). A prospective cohort study of 48 tumors with multiple molecular features found that the clinical and pathological characteristics of dMMR and p53-abn subtype tumors fell between those of pure dMMR and pure p53-abn subtype tumors (57).

The clinical management of tumors with multiple molecular features currently faces numerous uncertainties. On the one hand, although the current staging classification rules provide an operational framework, prospective validation is lacking regarding whether their prognostic assessments are fully equivalent to those for tumors with a single molecular feature. In particular, for the relatively common combination of dMMR and p53-abn subtype, controversy remains regarding whether its actual prognosis is closer to that of dMMR or p53-abn subtype (59). On the other hand, although the FIGO 2023 staging system has incorporated molecular subtyping into its staging modification framework, there is currently no unified clinical standard defining the criteria for upstaging or downstaging tumors with multiple molecular features. Future prospective studies are needed to address these challenges in the clinical management of tumors with multiple molecular features.

In summary, a comparison of the core characteristics of the four molecular subtypes is presented in **Table 1**.

**Table 1:** Core feature comparison table of molecular subtypes of endometrial cancer.

Molecular subtype	Corresponding TCGA classification	Key driver alterations	Molecular features	Typical prognosis	Current clinical implications
POLE-mutant (POLEmut)	POLE ultra-mutated	Pathogenic mutations in the exonuclease domain of the *POLE* gene	Ultra-high tumor mutational burden (TMB), hypermutator phenotype	Excellent, rare recurrence or disease-related death	De-escalated therapy (consider omitting adjuvant treatment)
Mismatch Repair-Deficient (dMMR)	Microsatellite Instability-High (MSI-H)	Functional loss of MMR genes (*MLH1, MSH2, MSH6, PMS2)*	High microsatellite instability (MSI-H), high tumor mutational burden (TMB)	Intermediate (between POLEmut and p53abn)	Immune checkpoint inhibitors (e.g., Pembrolizumab)
p53 abnormal (p53abn)	Copy Number High (CNH)	Pathogenic *TP53* mutations	High somatic copy number alterations, chromosomal instability	Poor, aggressive, accounts for >50% of deaths	Intensive adjuvant therapy (e.g., platinum-based chemotherapy)
No Specific Molecular Profile (NSMP)	Copy Number Low (CNL)	No specific *POLE*, *MMR*, or *TP53* alterations; frequent *PIK3CA, CTNNB1, KRAS* mutations	Low copy number variation, microsatellite stable (MSS), lower mutational burden	Intermediate (between POLEmut and p53abn)	Hormone therapy (especially for young patients) or conventional adjuvant therapy

## 4 Diagnostic strategies for molecular subtyping and their clinical integration

### 4.1 Molecular testing and multidisciplinary integration

In clinical practice, molecular subtyping of EC typically follows a stepwise analytical approach (**Figure 1**): first, *POLE* gene mutations are detected; second, MMR protein function is assessed by immunohistochemistry (IHC); and finally, p53 protein expression status is analyzed. If none of these abnormalities are detected, the case is classified as the NSMP subtype (60). Whole exome sequencing (WES) can serve as a single test approach for identifying different molecular subtypes and has good potential for clinical application (61). Combining next generation sequencing (NGS) with the ProMisE classifier also demonstrates high concordance and can be used for molecular subtype determination (62). Currently, IHC remains the cornerstone of initial evaluation, primarily for assessing MMR and p53 status. When combined with NGS, it can further improve the accuracy of molecular subtyping (63, 64). However, whether test results can reliably guide clinical decision making depends not only on the testing technology itself but also on the standardization of specimen processing, testing quality, and result interpretation (65). Not all *POLE* variants should be considered indicative of the POLE-mut subtype; only mutations in the exonuclease domain with clear pathogenic significance should be classified as this subtype (66). Following the detection of MLH1/PMS2 deficiency by MMR immunohistochemistry, further testing for MLH1 promoter methylation is required to distinguish sporadic dMMR subtype cases from those associated with Lynch syndrome (67). p53 immunohistochemistry must be interpreted strictly according to established criteria to avoid misinterpretation that could lead to bias in risk stratification and treatment decision making (68).

**Figure 1:**
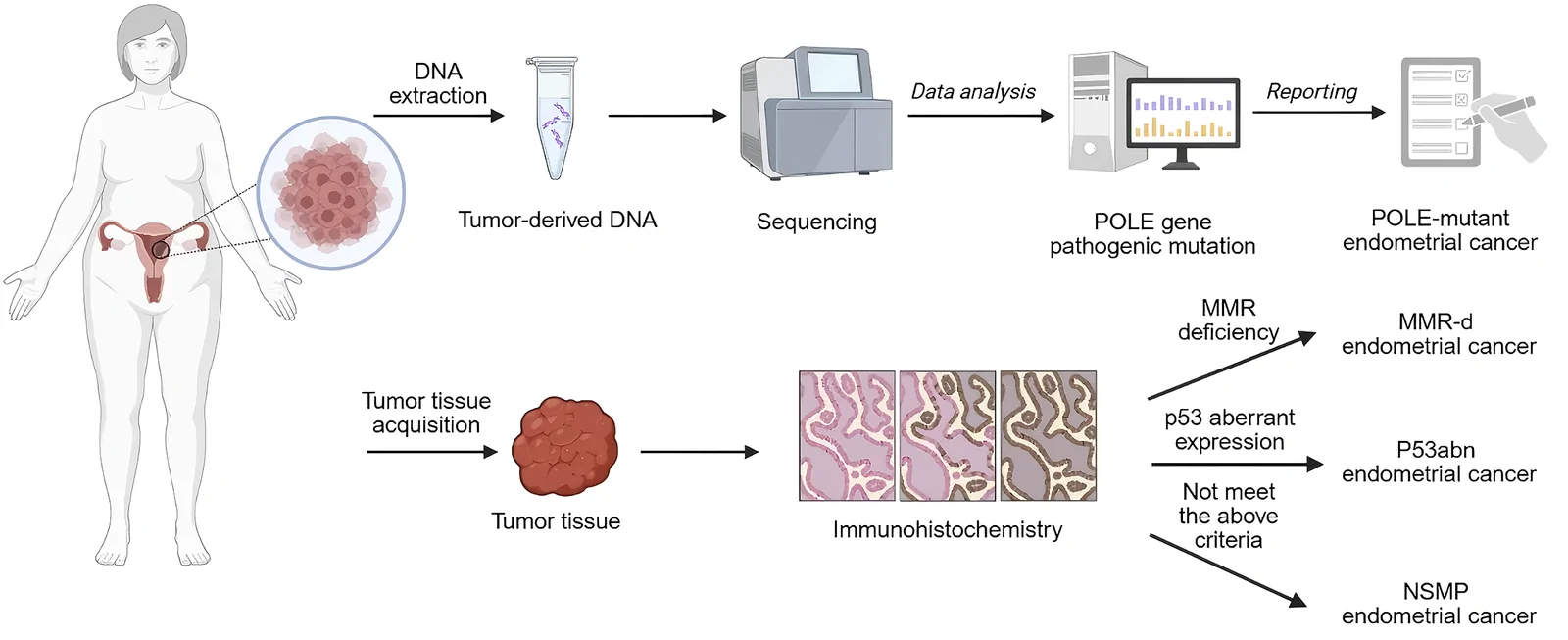
Stepwise algorithm for molecular classification of endometrial cancer.

Therefore, to ensure the accurate and effective implementation of molecular subtyping in clinical practice, the results should undergo comprehensive evaluation by a multidisciplinary team (MDT) comprising pathologists, molecular biologists, geneticists, radiologists, gynecologic oncologists, and medical oncologists, among others (11). After completing the histological diagnosis and MMR and p53 immunohistochemistry, the pathology department should perform *POLE* sequencing and, when indicated, MLH1 promoter methylation testing according to a predefined algorithm, ultimately generating a diagnostic report that integrates histological and molecular information (3, 10). During MDT discussions, molecular subtyping should be interpreted in conjunction with FIGO stage, histological type, tumor grade, lymphovascular space invasion, and patient related factors such as age and comorbidities. Treatment plans should not be determined independently of the broader clinical context (10, 11). For tumors exhibiting characteristics of multiple molecular subtypes simultaneously, a comprehensive assessment should consider both the reliability of the test results and the clinical significance of the features associated with each subtype. Through standardized testing, result verification, and MDT integration, classification errors can be reduced, enabling more reliable incorporation of molecular information into decision making regarding surgical treatment, adjuvant therapy, and systemic therapy (69).

### 4.2 Integrated diagnosis combining molecular subtyping and clinicopathological information

Molecular subtyping does not replace histological type, grade, depth of myometrial invasion, or lymphovascular space invasion (LVSI). Rather, it integrates the intrinsic biological characteristics of the tumor with traditional clinicopathological information. The FIGO 2023 staging system is based on this concept, combining molecular subtyping with histology and anatomical staging. For example, patients with stage I or II tumors classified as the POLE-mut subtype are downstaged to stage IA, whereas those with p53 abnormalities and myometrial involvement are upstaged to stage IIC. dMMR and NSMP typically do not alter the FIGO stage on their own (10). Consequently, the same anatomical stage may correspond to different prognostic risks and treatment decisions depending on molecular subtype, and molecular subtype results cannot be interpreted independently of tumor extent and other pathological risk factors.

This concept was further reinforced in the 2025 ESGO-ESTRO-ESP guidelines. The new guidelines incorporate FIGO 2023 staging, histology, LVSI, and other clinical and pathological factors into risk stratification and further refine risk categories for patients with NSMP and dMMR subtype. For example, low grade, ER positive NSMP subtype may be classified into a lower risk group at specific stages (11).

In comparison, the NCCN currently also emphasizes molecular testing but places greater emphasis on integrated molecular and genomic assessment. The NCCN 2026 edition explicitly states that the implementation of molecular testing should be tailored to the resources of the healthcare facility and the capabilities of the multidisciplinary team (MDT). Furthermore, testing can be performed on initial biopsy specimens, diagnostic curettage specimens, or final hysterectomy specimens (69). Therefore, the NCCN framework does not simply replace histology with a specific molecular subtype but emphasizes that molecular results, together with morphology, staging, and clinical factors, collectively form the basis for treatment decisions.

The ESMO pathway emphasized the link between molecular testing and treatment prognosis at an earlier stage. The 2022 ESMO guidelines recommend combined MMR and p53 immunohistochemistry with POLE testing for all endometrial cancers, while retaining traditional pathological variables such as histological type, FIGO grade, depth of myometrial invasion, and LVSI. In advanced or recurrent disease, MMR status in particular serves as a key molecular basis for selecting systemic therapy (67). Currently, some differences remain among guidelines regarding specific subgrouping and treatment recommendations. However, the overall framework is becoming increasingly consistent, and final clinical decisions still require consideration of tumor stage and other pathological risk factors.

### 4.3 Evaluation and management of hereditary endometrial cancer

Because Lynch syndrome associated EC lacks sufficient specificity in terms of age at onset, clinical presentation, and histology, screening based solely on family history, age at onset, or histological features may result in missed diagnoses. Therefore, an increasing number of guidelines currently support universal mismatch repair (MMR) testing for newly diagnosed endometrial cancer (70, 71). MMR immunohistochemistry is relatively simple to perform and can suggest potential pathogenic genes based on specific protein deficiency patterns. It is currently the primary method for clinical screening of Lynch syndrome associated endometrial cancer, while microsatellite instability testing can serve as an alternative or supplementary approach (70, 72). Universal testing does not mean that all patients undergo germline sequencing directly. Rather, a stratified strategy is adopted in which tumor MMR testing is performed first, followed by MLH1 methylation testing and finally selective germline testing, thereby improving detection rates while controlling testing costs (73, 74). Studies have shown that the sensitivity of traditional SGO clinical criteria for identifying potential Lynch syndrome is only 32.6%, suggesting a significant risk of missed diagnoses with selective testing (70). For tumors with combined MLH1/PMS2 deficiency, *MLH1* promoter methylation testing should be performed first. A positive result typically supports a sporadic tumor, whereas patients with no detected methylation and a deficiency in MSH2, MSH6, or PMS2 should undergo genetic counseling and further consideration of germline testing, which typically includes pathogenic variants in *MLH1, MSH2, MSH6, PMS2*, and *EPCAM* (71, 73, 75). Once a germline pathogenic variant is confirmed, management should extend beyond the patient’s own treatment to include long term surveillance for Lynch syndrome associated cancers and family risk management (71, 75). It is important to note that the dMMR molecular subtype is not synonymous with Lynch syndrome, as the majority of dMMR endometrial cancers are caused by sporadic mechanisms such as tumor acquired *MLH1* promoter methylation (71, 76). In a population based screening study, 27% of 233 patients exhibited abnormal MMR protein expression, but ultimately only 2.1% were diagnosed with Lynch syndrome based on germline testing (76). Therefore, dMMR is more suitable as an entry point for genetic risk assessment rather than as a basis for diagnosing Lynch syndrome. Clinically, the decision to proceed with germline testing should be made based on a combination of the MMR deficiency pattern and *MLH1* methylation results (70, 71, 76). Tumor molecular subtyping and genetic testing serve different clinical purposes: the former focuses on the molecular characteristics and clinical significance of the tumor itself, while the latter is used to determine whether a patient carries heritable pathogenic variants and to assess familial risk. The two are interrelated but not interchangeable (71).

### 4.4 Bottlenecks and challenges in clinical application

Molecular subtyping has gradually been integrated into clinical practice, but its widespread adoption in the real world remains limited by various factors. A multicenter survey showed that more than 90% of respondents considered *POLE*, MMR, and p53 testing important for clinical decision making, but only 24.2% considered *POLE* testing to be readily available. Cost was identified as a major barrier to the widespread adoption of molecular subtyping (77). The widespread adoption of molecular subtyping depends not only on the testing technology itself but also on health insurance reimbursement policies and the capacity of the healthcare system to bear the ongoing costs of testing. Surveys conducted in several Eastern European countries indicate that access to molecular subtyping is severely limited by inadequate reimbursement mechanisms and insufficient laboratory infrastructure (71).

The clinical implementation of molecular subtyping must be tailored to local conditions, with a stratified testing strategy rather than requiring all patients to undergo exactly the same testing process. In low and middle income countries and other resource limited regions, priority should be given to tests that are simple to perform and cost effective, such as MMR and p53 immunohistochemistry, while *POLE* sequencing should be centralized in regional hub laboratories or referral laboratories. Recent international expert consensus on resource limited settings also recommends prioritizing tests according to the impact of their results on clinical decision making (78, 79).

Standardization of laboratory testing and quality assurance are equally critical components of clinical implementation. Differences among centers in tissue selection, immunohistochemical staining, and interpretation of p53 and MMR results may lead to inconsistencies in molecular subtype classification. In a cross laboratory study, ProMisE demonstrated high overall consistency, but some inconsistencies were primarily attributable to weak p53 staining and errors in MMR protein interpretation, suggesting that technical quality control, pathologist training, and repeat testing when necessary are indispensable (80). Therefore, establishing standardized operating procedures, clarifying interpretation criteria, and fostering interdisciplinary collaboration are essential measures to ensure the accuracy of molecular subtyping (80, 81).

Pathology department workflows and turnaround times also directly influence whether molecular results can effectively inform clinical decision making. Studies have shown that initiating molecular testing at the endometrial biopsy stage allows 82.0% of cases to receive results before multidisciplinary discussions, whereas this proportion drops to only 8.4% when testing begins at the hysterectomy specimen stage. In this study, a median turnaround time of 18 working days was achieved through centralized testing (81). Another study demonstrated that process optimization can maintain NGS turnaround times at approximately 10 to 14 days while reducing implementation disparities across different healthcare institutions (82).

Similarly, the implementation of molecular subtyping technologies has raised numerous ethical and social issues. If access to these technologies is unequal among patients, the high cost of implementation may exacerbate the uneven distribution of healthcare resources (83). The large amounts of genomic data generated by germline testing, AI models, and other methods also raise concerns regarding privacy protection, informed consent, and algorithmic bias (84). Protecting the privacy of patients’ genomic information and ensuring data security are of paramount importance. Existing research indicates that the distribution of molecular subtypes varies across different racial populations, suggesting that research and clinical applications related to molecular subtyping should fully account for population differences to avoid further exacerbating health inequalities due to disparities in access to evidence and technology (85).

In summary, although molecular subtyping holds substantial potential for clinical practice, its full implementation still faces multiple challenges. Addressing these concerns requires the joint efforts of policymakers, healthcare providers, and all sectors of society. By reducing costs, improving accessibility, shortening turnaround times, and standardizing methodologies, among other measures, the clinical value of molecular subtyping can be better realized.

## 5 Treatment strategies based on molecular subtyping of endometrial cancer

### 5.1 Individualized decision-making for adjuvant therapy

Individualized treatment based on molecular subtyping is becoming the standard treatment regimen (**Figure 2**).

**Figure 2:**
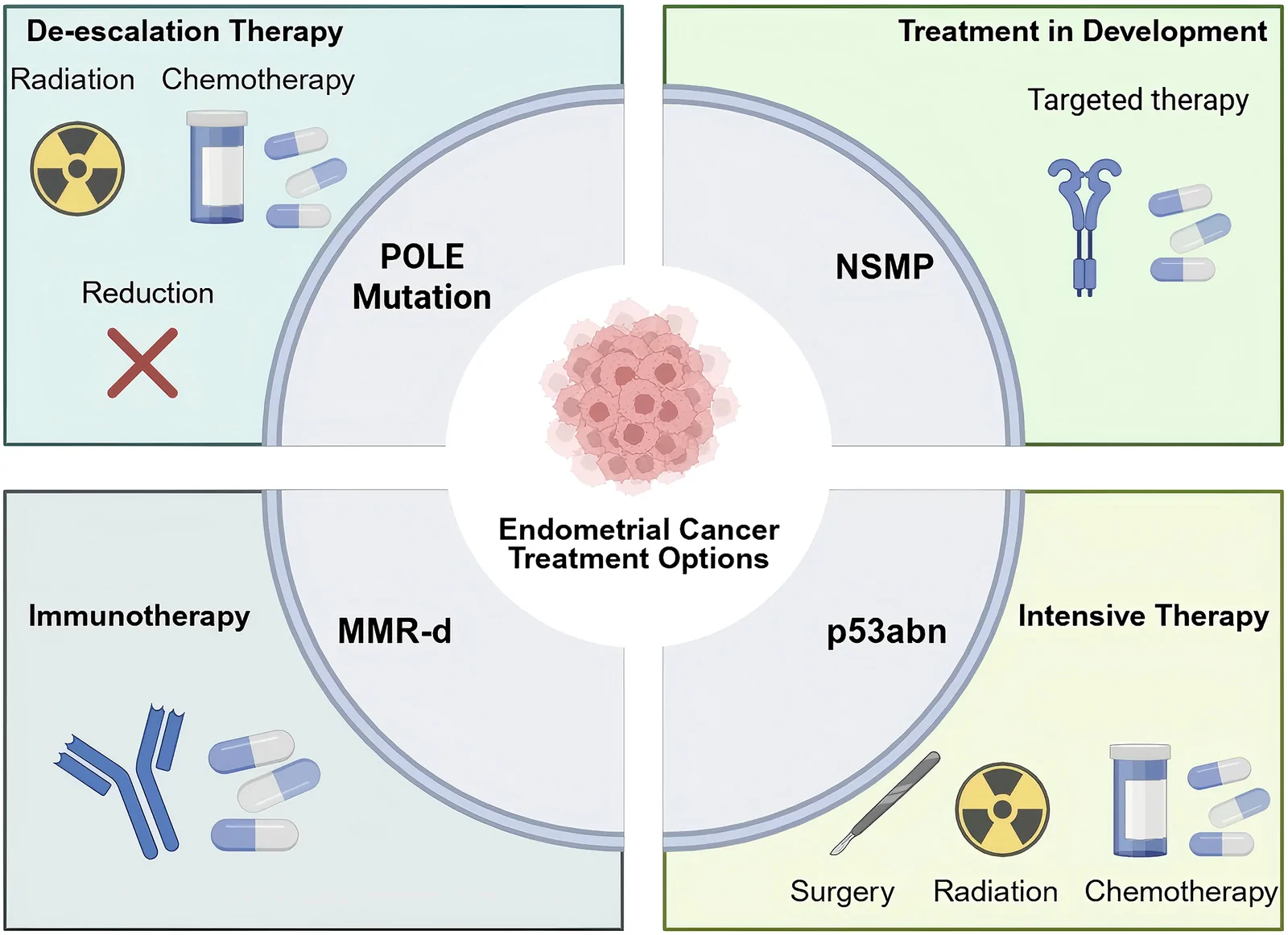
Personalized treatment plans for endometrial cancer based on molecular subtyping.

The POLE-mut subtype EC exhibits a significant hypermutation phenotype and a favorable prognosis. Its potential for treatment de-escalation stems primarily from a lower risk of recurrence rather than from the molecular subtype itself (12, 86). Molecular subgroup analysis from the PORTEC-3 trial showed that the 5 year recurrence free survival rate among patients with high risk endometrial cancer of the POLE-mut subtype reached 98%, while the addition of chemoradiotherapy did not demonstrate a clear benefit compared with radiotherapy alone, suggesting that this subtype may have the potential to avoid excessive adjuvant therapy (12). Subsequently, some studies have begun to explore reducing or omitting adjuvant therapy in carefully selected patients with POLE-mut subtype. However, the current evidence primarily comes from small sample retrospective studies, and randomized trials specifically targeting patients with POLE-mut subtype remain lacking (36). The recently published PORTEC-4a Phase III study further demonstrates the feasibility of using integrated molecular risk profiles to guide adjuvant treatment decisions in patients with high-intermediate-risk early stage endometrial cancer. Among these patients, those with POLE-mut subtype may undergo observation (13). However, because this study evaluated adjuvant treatment strategies guided by integrated molecular risk profiles, it does not directly imply that all patients with POLE-mut subtype can safely omit adjuvant therapy (13). Consequently, treatment de-escalation for patients with POLE-mut subtype has progressed from retrospective exploration to prospective validation. However, a unified standard treatment strategy has not yet been established. The POLE-mut subtype BLUE trial within the RAINBO project is further evaluating the safety of completely omitting adjuvant therapy or using radiotherapy alone in different risk contexts. Its results will provide more direct evidence regarding whether this strategy can be incorporated into standard practice (14). At this stage, the clinical value of POLE-mut subtype is primarily reflected in prognostic prediction, while evidence regarding their predictive value for treatment response remains insufficient. The POLE-mut subtype cannot yet serve as the sole basis for exempting all patients from adjuvant therapy. Clinical decision making must still involve a comprehensive assessment incorporating tumor stage, histological type, and other pathological risk factors.

The p53-abn subtype typically exhibits more aggressive biological behavior and poorer clinical outcomes, a phenomenon particularly pronounced in high risk endometrial cancer. A molecular subgroup analysis of the PORTEC 3 trial showed that patients with the p53-abn subtype derived greater benefit from chemoradiotherapy than those with other subtypes. Their 5 year recurrence free survival rates were 59% and 36% in the chemoradiotherapy and radiotherapy groups, respectively (12). These results support the consideration of intensified treatment for patients with the p53-abn subtype. Longer term 10 year follow up further supports this trend. The 10 year RFS rates for patients with p53 abnormal endometrial cancer in the CTRT and RT groups were 52.6% and 37.0%, respectively (HR 0.42, 95% CI 0.24 to 0.74), and the 10 year OS rates were 52.7% and 36.6%, respectively (HR 0.52, 95% CI 0.30 to 0.91) (87). Therefore, current evidence supports p53-abn status as an important molecular marker for identifying high risk patients and suggests its potential value in treatment stratification. However, it does not support the conclusion that all patients with p53-abn subtype status should receive treatment intensification of equal intensity. Current evidence for treatment intensification primarily stems from *post hoc* molecular subgroup analyses in high risk populations rather than from trials specifically designed for patients with p53 abnormalities. Given the limited sample sizes and lack of statistically significant interactions, this evidence cannot yet be considered definitive grounds for changing clinical practice (14). The ongoing RAINBO p53-abn subtype RED trial is further investigating whether patients with p53 abnormalities can benefit from a molecularly guided intensification strategy by adding olaparib after adjuvant chemoradiotherapy (14). Therefore, the decision to intensify treatment for patients with p53 abnormalities should still be made in conjunction with tumor stage, histology, and other risk factors and requires further validation through prospective studies.

The NSMP subtype is the most heterogeneous of the four molecular subtypes, and there is currently a lack of clearly defined subtype specific targets that can directly guide adjuvant therapy (88). Specific details have been discussed in previous sections. Therefore, the clinical value of the NSMP subtype currently lies more in further refining risk assessment based on traditional clinical and pathological factors than in directly selecting a specific treatment based on this subtype alone. This point is particularly important because, although markers such as *CTNNB1*, L1CAM, *ARID1A*, and ER/PR have potential prognostic or therapeutic significance, most of the evidence still comes from observational studies and is insufficient to establish mature, subtype specific adjuvant treatment regimens (88, 89).

### 5.2 Immunotherapy

The dMMR/MSI-H subtype of EC exhibits a high mutation burden and a strong capacity for neoantigen generation, making it highly sensitive to immune checkpoint inhibitors. Its core clinical value has therefore gradually shifted from prognostic stratification toward treatment prediction (56). Pembrolizumab has been approved for the treatment of advanced MSI-H/dMMR subtype EC (90). Two Phase III randomized trials, RUBY and NRG-GY018, further confirmed that adding immune checkpoint inhibitors to standard carboplatin and paclitaxel chemotherapy significantly improves progression free survival in patients with advanced or recurrent endometrial cancer, with particularly pronounced benefits observed in the dMMR subtype subgroup (15, 16). The RUBY study demonstrated that dostarlimab combined with chemotherapy significantly improved progression free survival in the overall population and produced a more pronounced therapeutic effect in the dMMR/MSI-H subtype population (15). The NRG-GY018 study showed that pembrolizumab combined with chemotherapy improved progression free survival in both the dMMR and pMMR subtype subgroups, but the benefit was greater in the dMMR subtype subgroup (16). Therefore, dMMR has become one of the most clearly established biomarkers for translating prognostic information into treatment decisions among the four molecular subtypes. However, its prognostic value in adjuvant therapy remains much less clear. A molecular subgroup analysis of the PORTEC-3 trial showed that patients with dMMR subtype high risk endometrial cancer did not derive a clear benefit from adjuvant chemotherapy. There was no significant difference in 5 year recurrence free survival between chemoradiotherapy and radiotherapy alone (12). Meanwhile, whether patients with early stage dMMR subtype endometrial cancer can benefit from adjuvant immunotherapy remains an evidence gap requiring further high level evidence. At the same time, both the NRG-GY018 and DUO-E trials suggest that patients with pMMR subtype endometrial cancer may also derive progression free survival benefits from combination immunotherapy strategies. However, this benefit cannot be simply attributed to MMR status and is not yet sufficient to indicate the identification of new molecular predictive biomarkers (16, 91). Therefore, the most clearly established clinical value of dMMR subtype currently lies primarily in predicting response to immunotherapy in advanced or recurrent disease, while its role in early adjuvant therapy remains to be further clarified. At this stage, clinical decision making should still involve a comprehensive assessment incorporating factors such as FIGO stage, histological type, tumor grade, and lymphovascular space invasion.

A p53-abn phenotype does not imply that immunotherapy is ineffective. A prespecified subgroup analysis from the RUBY trial showed that the benefit of dostarlimab combined with chemotherapy was not limited to the dMMR subtype population. A therapeutic effect was also observed in patients with a p53-abn phenotype (15). However, because these results primarily stem from subgroup analyses, they are not yet sufficient to demonstrate that the p53-abn subtype phenotype itself is a predictive biomarker for immunotherapy.

Previous studies have found that the POLE-mut subtype are associated with significantly enhanced antitumor immune responses, and case reports have shown that patients with *POLE* mutations exhibit significant responses to pembrolizumab (92). However, high immunogenicity does not equate to a proven need for immunotherapy. The POLE-mut subtype itself typically carries a favorable prognosis. Therefore, randomized trial evidence supporting the clinical benefit of proactively adding immunotherapy in early stage disease remains lacking. Currently, the value of immunotherapy in this context is primarily an area of interest for biological and translational research.

### 5.3 Emerging biomarkers and treatment prediction

Targeted therapy for endometrial cancer has made progress based on molecular subtyping. Given the high prevalence of abnormalities in the PI3K signaling pathway in endometrial cancer, including *PIK3CA* mutations and *PTEN* loss, inhibitors targeting PI3K, AKT, and mTOR are currently under investigation (93, 94). Antiangiogenic agents, such as lenvatinib in combination with pembrolizumab, have demonstrated efficacy in the treatment of advanced endometrial cancer (90). In addition, poly ADP ribose polymerase inhibitors have demonstrated potential clinical value in a subgroup of patients with endometrial cancer harboring homologous recombination defects (14, 91).

Antibody drug conjugates (ADCs) are currently among the most promising emerging therapeutic approaches, with HER2 serving as a well-defined actionable therapeutic target. The DESTINY PanTumor02 Phase II trial demonstrated that trastuzumab deruxtecan (T-DXd) achieved a high objective response rate in patients with HER2 expressing endometrial cancer, with the most pronounced benefit observed in the HER2 IHC 3+ subgroup (95). A gynecologic oncology analysis published in 2026 further supported the antitumor activity of T-DXd in HER2 expressing endometrial cancer (96).

## 6 Future prospects

Current molecular subtyping primarily relies on a single tissue sample, which reflects the tumor’s immediate molecular characteristics but may not fully capture intratumoral cellular heterogeneity or molecular changes during disease progression and treatment (8, 11). With the development of emerging technologies, our understanding of the biology and dynamic changes of endometrial cancer is evolving. Single cell sequencing and spatial transcriptomics hold promise for characterizing tumors and their microenvironments at the levels of cellular composition and spatial distribution, providing new perspectives for explaining biological and clinical differences among patients with the same molecular subtype (97, 98). Multiomics integration may further reveal the molecular characteristics of highly heterogeneous subgroups such as NSMP subtype. However, whether these new findings can lead to stable and clinically meaningful reclassification remains to be validated in prospective studies (99).Meanwhile, circulating tumor DNA (ctDNA) and liquid biopsy offer new possibilities for dynamically monitoring disease progression. Existing studies suggest that persistent detection of ctDNA after surgery is associated with an increased risk of recurrence, and some molecular abnormalities may even appear earlier than radiological or clinical manifestations (100). However, early detection of molecular recurrence does not necessarily translate into improved survival outcomes. There is currently insufficient prospective evidence to determine whether ctDNA can be further used to guide treatment intensification, treatment de-escalation, or treatment selection for recurrence (100).In terms of improving the accessibility of molecular subtyping, artificial intelligence in pathology, digital pathology, and MRI radiomics have also shown potential for predicting certain molecular characteristics using routine histological sections or imaging data (101, 102). However, these methods are currently limited by factors such as sample size, differences in testing platforms, model generalizability, and insufficient external validation. They are therefore more suitable as adjunctive or screening tools for standard molecular testing rather than as direct replacements for existing tests. From this perspective, the future direction of molecular subtyping is not to establish increasingly complex and refined classification systems, but rather to achieve a more continuous understanding of tumor heterogeneity, disease progression, and treatment response. By ensuring accuracy and reproducibility while improving the accessibility of testing (103), molecular subtyping may ultimately provide more effective guidance for clinical decision making.

## 7 Conclusion

The emergence of molecular subtyping has transformed the risk stratification system for EC. From the landmark discovery of molecular subtypes in the TCGA to the validation of clinically implementable strategies such as ProMisE and, ultimately, their integration into international guidelines, this evolution marks a shift in the diagnosis and treatment of EC from an empirical, morphology based approach toward a precision medicine model grounded in molecular biology.

The value of molecular subtyping for prognostic risk assessment has been widely validated. However, the strength of evidence supporting clinical treatment decisions remains significantly uneven across molecular subtypes. The dMMR subtype has successfully transitioned from molecular stratification to treatment prediction for immunotherapy in advanced or recurrent disease, making it the subtype with the most clearly defined clinical utility among the four subtypes. The prognostic advantage of the POLE-mut subtype has been well validated through retrospective cohorts and subgroup analyses of clinical trials. However, treatment de-escalation strategies remain in the exploratory phase. The identification of high risk patients within the p53-abn subtype is widely recognized, but the patient population that would benefit from treatment intensification requires further clarification through clinical trials. As a residual category defined by exclusion, the substantial internal heterogeneity of the NSMP subtype is gradually being elucidated through markers such as hormone receptor status, L1CAM, *CTNNB1*, *ARID1A*, and HLA-I deficiency. However, the path from biomarker discovery to clinical validation remains long. The emergence of tumors with multiple molecular features further challenges the conventional mutually exclusive four-subtype classification framework.

The root cause of this heterogeneity in the evidence lies in differences in study design across different stages of research. These differences have generally confined molecular subtyping to prognostic prediction rather than treatment prediction. Prospective trials such as RAINBO are expected to partially address this evidence gap, but as their results have not yet been published, current clinical practice still requires a balance between traditional pathological parameters and molecular information. The expansion of emerging technologies does not automatically bridge the evidence gap. Cutting edge methods such as spatial transcriptomics and liquid biopsy, if not supported by prospective validation linked to clinical outcomes, remain tools for more detailed characterization rather than tools for clinical decision making. The greatest challenge lies in the fact that, even as technologies mature, disparities in global access remain difficult to overcome. The ultimate value of molecular subtyping lies not in identifying more subgroups, but in ensuring that every patient, regardless of geographic location or economic status, can benefit from molecular information.

The focus in the future should not be on discovering more biomarkers, but on obtaining more prospective evidence to guide clinical decision making, driving the translation of molecular discoveries into treatment strategies, and ultimately improving patient outcomes. With further advancements in molecular testing technologies, clinical research, and multidisciplinary diagnosis and treatment, molecular subtyping is expected to evolve from a risk stratification tool into a treatment decision making tool, providing patients with endometrial cancer with more precise and clinically valuable diagnostic and treatment strategies.
